# Gut Microbiota and Lymphocyte Subsets in Canine Leishmaniasis

**DOI:** 10.3389/fvets.2022.868967

**Published:** 2022-07-13

**Authors:** Sara Meazzi, Stefania Lauzi, Valeria Martini, Riccardo Ferriani, Margherita Peri, Sergio Aurelio Zanzani, Alessia Giordano

**Affiliations:** ^1^Department of Veterinary Medicine and Animal Sciences, University of Milan, Lodi, Italy; ^2^Ospedale Veterinario San Francesco, Milan, Italy

**Keywords:** *Leishmania* spp., fecal microbiome, leukocytes, flow cytometry, bacteria

## Abstract

Gut microbiota seems to interact with immune system. Canine leishmaniasis pathogenesis and severity of disease lean on the host immunity, but there is no information in literature about gut microbiota in infected animals. Thus, this study aims to compare the microbiota composition and leukocyte subset of healthy dogs with those of asymptomatic dogs exposed to *Leishmania* spp. and dogs with clinical leishmaniasis. Thirty-nine dogs were enrolled and grouped into three groups: healthy, exposed asymptomatic and infected symptomatic for *Leishmania* spp. Flow cytometry on whole blood evaluated the prevalence of CD4, CD5, CD8, CD11b, CD14, and CD21 positive cells. Gut microbiota was investigated using a next generation sequencing (NGS) technique. Firmicutes resulted significantly more abundant in the healthy dogs compared with the other two groups. Conversely, Proteobacteria were more abundant in symptomatic dogs. Even in rarest phyla comparison some significant differences were found, as well as in comparison at classes, order, family and genus levels. The symptomatic group had lower concentration of all the lymphocyte classes (CD5, CD21, CD4, CD8) compared to the other groups. A lower abundance of Firmicutes is reported in literature in diseased animals compared to the healthy ones and this is in agreement with the results of this study. Increased Proteobacteria in sick animals could suggest a dysbiosis status, even without distinct gastrointestinal signs. The leukocyte classes results indicate a decreased Th1 response in symptomatic dogs. Studies also investigating the cytokine response could deepen the knowledge on the pathogenesis of canine leishmaniasis.

## Introduction

The gut microbiota is the ensemble of all the bacteria that live into the digestive tract (GI) (1). It plays different roles in the host homeostasis, contributing to food degradation and energy harvest. Moreover, some bacteria can produce metabolites needed for nutrition of the enterocytes. Finally, it acts as a physical obstacle against pathogens, and it interacts with the immunity (2). Furthermore, gut microbiota may also influence gastrointestinal tract defenses at different levels both helping the mucous layer formation (3) and inducing the generation of regulatory T cells (Treg), needed for the symbiotic relationship between bacteria and host (4). The association between the host immunity and the gut microbiota appears to be extended also to organs other than the intestine (4). The microbiota has a crucial role in the secondary lymphoid tissues development, as well as in CD4+ and CD8+ T lymphocytes, Treg, T helper 17 (Th17) cells and B lymphocytes differentiation and activity (5). The impaired immune response observed in animal models with manipulated microbiota, highlights the importance of the gut microbiota for the host. In germ-free mice, the absence of commensal bacteria has been associated to a reduced development not only of gut-associated lymphoid tissues (GALT), but also systemic ones. Furthermore, a decrease in circulating T and B lymphocytes together with a of Th1/Th2 imbalance was observed in the germ-free mice (6–8). Gut microbiota assessment could be performed on different type of specimens. Even though results obtained on stool samples are mostly representative of the distal gut, this kind of samples could be very easily obtained. In the evaluation of microbiota composition, factors such as antibiotic treatments, diet, and other environmental factors should be taken in account both in people and animals (4, 9–12). Several veterinary medicine studies evaluate the gut microbiota differences during dysbiosis (caused by different conditions) (13, 14). Specifically, microbiota composition imbalances may occur during inflammatory or infectious diseases (15). *Leishmania* spp. transmitted by a phlebotomine sand fly vector is the cause of a severe systemic condition namely canine leishmaniasis (16, 17). Both the protozoan and the host immunity are responsible for the development of the clinical signs of disease (17): *Leishmania* spp. is generally deposited in the skin by the sand fly during the blood meal; here it may be destroyed by the host immune system (self-limited infection), or it may be confined in the skin or lymph nodes without any clinical sign (asymptomatic infection), or it may diffuse systemically causing either symptomatic or asymptomatic infection depending on the host immunity (18). During *Leishmania* infection, cell-mediated immunity is pivotal for a successful response (19) and different subsets of T lymphocytes may influence the host predisposition for the clinical form of the diseases (16). In literature, a decrease of CD4+ Th lymphocytes and of CD8+ T cells in peripheral blood (20–22) has been reported in susceptible dogs. For several years, Th1 (cell mediated) was considered to be protective against the disease, contrarily Th2 (humoral response) was associated with (23) canine leishmaniasis predisposition. Nevertheless, it seems that infected dogs may mount both Th1 and Th2 responses, even if the Th1 is the predominant phenotype in resistant dogs (18). Given the pivotal role of immune response in the pathogenesis of canine leishmaniasis, the aims of this work were to evaluate the gut microbiota variations and the different leukocyte classes in canine peripheral blood of animals with clinical signs of leishmaniasis compared to dogs exposed to *Leishmania* spp. but asymptomatic and to healthy control unexposed dogs.

## Materials and Methods

### Dogs/Caseload

All the 39 dogs enrolled in this prospective study were referred to the Veterinary Teaching Hospital of The University of Milan or to the private veterinary clinic Ospedale S. Francesco of Milan (Italy) for routine wellness visit or diagnostic purpose and each owner signed an informed consent for the diagnostic sampling performed during those visits. Therefore, according to the Ethical Committee decision of the University of Milan, residual aliquots of samples or tissues collected during routine visit, can be employed for research purposes without any supplementary authorization (EC decision 29 Oct 2012, renewed with the protocol no. 02-2016). Dogs were considered eligible for inclusion when they met the following inclusion criteria: (1) no antibiotic or probiotic treatment for at least 3 months prior to inclusion, (2) no gastroenteric clinical signs for at least 2 weeks prior to inclusion, (3) negative result of the microscopic fecal examination for helminths and the detection of coproantigen of Giardia duodenalis by ELISA, (4) negative serological result using the SNAP® 4Dx® test (IDEXX) detecting *Dirofilaria immitis* antigen and antibodies against *Anaplasma phagocytophilium, Anaplasma platys, Borrelia burgdoferi, Erlichia canis* and *Erlichia ewingii* (5) fed on commercial food (6) no previous vaccination against *Leishmania* spp. Once enrolled, based on both screening analyses (that includes qPCR and IFAT for *Leishmania* spp.) and history, the dogs were further classified into three groups: healthy (H); *Leishmania* spp. exposed but asymptomatic (E_A); infected with *Leishmania* spp. and with the presence of clinical signs consistent with canine leishmaniasis (S) according to Canine Leishmaniasis Working Group guidelines (17). Specifically, in the H group dogs have no clinical signs or laboratory abnormalities and negative results for both IFAT (immunofluorescence antibody test) and qPCR for *Leishmania* on whole blood. All the dogs belonging to this group lived in a non-endemic area. E_A group included dogs with an absence of clinical signs nor clinicopathological abnormalities that suggest the presence of leishmaniasis or other diseases, but with weakly positive IFAT result (≤ 1:80) and negative *Leishmania* qPCR on blood samples. The history of these dogs always included origin and living in an endemic area and a past history of strong positive IFAT result for *Leishmania* antibodies or previous clinical manifestation of canine leishmaniasis. Finally, the S group was composed by sick dogs that presented with clinical manifestation of leishmaniasis together with clinicopathological alterations, positive IFAT result and the presence of the parasite (identified by PCR on blood or lymph-node and/or bone marrow cytology).

### Sample Collection

At admission, for each dog 2 mL of whole blood were collected and subsequently divided into an EDTA tube and a plain tube (Venoject, Terumo Italia Srl, Rome, Italy). On EDTA samples, a complete blood cell count (CBC) was performed within 12–18 h, 200 μL were frozen at −20° for qPCR analyses and 0.5 mL were used for flow cytometric analyses. The serum tube was centrifuged at 2,500 × g for 5 min for serum harvesting. From each dog, at least 15 g of fresh feces were collected in one sampling and split into two aliquots: a fresh one for microscopic fecal evaluation and the detection of coproantigen of *Giardia duodenalis* by ELISA and another aliquot for the microbiota evaluation was immediately frozen at −20°. A sample of urine from each dog was collected by spontaneous micturition (at least 5 mL) and centrifuged at 1,250 × g for 5 min for supernatant harvesting and proteinuria evaluation.

### Screening Analyses

Screening analyses were performed to group the enrolled dogs. On EDTA samples a CBC was evaluated through the automated hematology analyser Sysmex XT-2000iV (Sysmex corporation, Kobe, Japan) together with a microscopical examination of stained blood smears. On serum, a routine biochemistry panel (glucose, creatinine, urea, total protein, albumin, alanine aminotransferase and alkaline phosphatase) was executed with the automated spectrophotometer BT3500 (Biotecnica instruments S.p.a, Roma, Italia). Agarose gel serum protein electrophoresis, that is considered an accurate test to diagnose canine leishmaniasis, was achieved through the use of the semiautomated instrument Hydrasis (Sebia Italia S.r.l., Bagno a Ripoli, Florence, Italy). The typical electrophoretic pattern for canine leishmaniasis includes hypoalbuminemia and increase in α2 and γ globulin (sometimes there is also an increase in the β2 fraction, with the so-called β-γ bridge). The decrease in A:G is considered one of the most sensitive test for canine leishmaniasis (24). On urine supernatant, using the same instrument employed for serum biochemistry, the urinary protein to creatinine (UPC) ratio was assessed. The immunofluorescence antibody test (IFAT) was used to evaluate the antibody titer against *Leishmania* thanks to the MegaFLUO® Leish commercial kit (Diagnostik Megacor, Hörbranz, Austria). *L. infantum* DNA identification using real time PCR (qPCR) was assessed on EDTA samples following the instruction provided by the manufacturer of the commercial kit used (NucleoSpin® Blood, Macherey-Nagel, Germania). DNA pre-analytical quality control targeting vertebrate 12S rRNA locus was performed on randomly selected EDTA samples (results not shown) (25). The qPCR used as a target *L. infantum* kinetoplast minicircle DNA conserved regions and was performed according to published protocol (26), with minor modifications, using the instrument QuantStudio3 (Applied Biosystem). The parasite load measure was considered the number of threshold cycle (CT), with a lower number of CT indicate a higher burden of *L. infantum*. The positive control was represented by a canine sample that was previously tested for *L. infantum* and resulted positive, whereas for the negative control was used water.

### Gut Microbiota Analyses

Microbial communities (Metabarcoding analyses) using sequencing of amplicon were investigated throughout the hypervariable genomic region (V3-V4 region 16SrRNA gene amplification), using an NGS approach on Illumina Platform. From 180 to 220 mg of each fecal sample the total amount of DNA was extracted following the instruction of the manufacturer and using the QIAamp DNA Stool Mini Kit (QIAGEN S.r.l., Milan, Italy). Using the Illumina 16srRNA protocol indexed NGS libraries were built. At first PCR was used to amplify template from a DNA sample using specific denatured primers targeting the 16S V3 and V4 region. One microliter of this product was evaluated for size (~550 bp) on a Bioanalyzer DNA 1000 chip (2100 Bioanalyzer with DNA 1000 Kit, Agilent, Santa Clara, CA, USA). Then, AMPure XP bead removed free primers and primers dimers (A63880I; Beckman Coulter Inc., Brea, CA, USA) allowing to complete a second PCR to attach dual index and Illumina sequencing adapters using the Nextera XT Index kit (Illumina, San Diego, CA, USA). Another cleaning step using AMPure XP bead was performed in order to prepare the final library for the quantification. The final library was diluted 1:50 and then on 1 μL was evaluated the sample size (~630 bp) using Bioanalyzer DNA 1000 chip already mentioned. The DNA concentration expressed in nM and based on the DNA amplicons size, was used to pool the obtained 60 libraries in equimolar concentration. Finally, the pooled library was sequenced using the Illumina Miseq technology in 2 × 300 bp run using 20% of PhiX library as control (27).

### Flow Cytometry Analyses

Leukocyte populations of CD4+, CD8+, CD5+, CD14+, CD11b+, and CD21+ cells were assessed using the flow cytometer BryCyte E6 (Mindray, Schengen, China). The obtained results were analyzed using a dedicated software (MRFlow, Mindray, Schengen, China) (27). Each sample was run on three tubes and each tube contained exactly 500 × 10^3^ cells (based on the formula 500/WBC applied on hematologic results). Twenty-five microliter of RPMI solution containing 0.2% fetal bovine serum (FBS) were added to decrease the non-specific antibodies binding. Then, 50 μL of the specific antibody dilution was added accordingly to the following scheme (see Table 1): tube 1 was used as negative control (no antibody); tube 2 contained antibody directed against CD5, CD8, and CD4; finally, tube 3 contained antibody directed against CD14, CD11b, and CD21. Each tube incubated at room temperature for at least 15 min. Then, an 8% ammonium chloride lysing solution was added to remove the erythrocytes and samples were washed through centrifugation (1,250 rpm for 8 min). The supernatant was thrown away and the pellet was resuspended in 500 μL of PBS and acquired by the flow cytometer. A first gate was set in a morphological scattergram (forward scatter, FSC-H, vs. side scatter, SSC-h) to keep out platelets and cellular fragments from the analyses. Subsequently, based on complexity and fluorescence, cell populations were analyzed. For each sample the percentage cell positive for CD5, CD8, CD4 (to evaluate the lymphocytes only the low complexity cells were included), CD14, CD11b, CD21 was registered. The neutrophils percentage was calculated through the following formula: %CD11b-%CD14. Finally, CD4+:CD8+ ratio was calculated. All the recorded percentages were then used, together with the leukocytes total number, to calculate the absolute number of each white blood cell class (27).

**Table 1:** List of the antibodies in use, their specificity and dilution.

**Antibody**	**Clone**	**Fluorocrome**	**Specificity**	**Producer**	**Dilution**
CD5	YKIX322.3	FITC	T lymphocytes	Serotec, Oxford, UK	1:400
CD8	YCATE55.9	PE	T cytotoxic lymphocytes	Serotec, Oxford, UK	1:35
CD4	YKIX302.9	AF-647	T helper lymphocytes, neutrophils	Serotec, Oxford, UK	1:50
CD14	TUK4	PE	Monocyte	Serotec, Oxford, UK	1:25
CD11b	M1/70	PE-cy5	Neutrophils, monocytes	eBioscience, San Diego, CA, USA	1:500
CD21	CA2.1D6	AF-647	B lymphocytes	Serotec, Oxford, UK	1:200

### Data Analyses

Microbiota analysis was based on *de novo* read counts that have been built using the addition of both the quality scores and sequence frequencies in a probabilistic noise model for nucleotide transitions. All the sequences were filtered and the chimerae removed. Then, the obtained data were compared to a standard database of bacteria, and they were labeled accordingly (28). The main step of the sequence analysis is the denoised and assembly of sequences into groups called Ribosomal Sequence Variants (RSVs) instead of the traditional Operational Taxonomic Units (OTUs). The workflow was based on software packages from the open-source Bioconductor project (28). At first the low-quality sequencing reads were removed and all the reads were trimmed to an adequate length. Afterward sequence variants were inferred and, following the steps already proposed by Callahan et al. (28), a sequence table (an analog, with a higher resolution, of the “OTU table”) was generated. All these phases were achieved through a DADA2 method that is based on a substitution error parameterized model that allow to distinguish possible sequencing errors from real biological variation (28). In order to correctly allocate the taxonomy, a naive Bayesian classifier method was used: it performed a comparison between sequence variants and a classified sequences training set (GreenGenes V13 gg_13_8_train_set_97). Alpha diversity was evaluated through Observed and Shannon metrics. Rarefaction Species curve was obtained using “rarecurve” function of Vegan Package (vegan: Community Ecology Package. R package version 2.5-4.). The alpha diversity differences among groups were assessed using a Kruskal–Wallis (followed, when significant results were obtained, by a *post-hoc* test) performed with Analyse-it Software for Microsoft excel. Beta diversity (that assess the between sample diversity) was evaluated using non-metric multidimensional scaling (NMDS) and principal coordinate decomposition (also known as classical scaling) of a distance matrix (PCOA) was used to graphically described the diversity between samples in a low-dimensional space based on Bray-Curtis dissimilarity matrix and Jaccard dissimilarity matrix. The phyloseq-format microbiome data was converted into a DESeqDataSet for Differential Abundance OTU call (29). Specifically, DESeq2 carried out for each OTU a hypothesis test to assess if results were considered enough to refuse the null hypothesis, namely that the difference observed was caused only by experimental variability. Differences in each leukocyte subset, both expressed as percentage or as absolute number, among the groups were evaluated using Kruskall-Wallis test (when statistical differences were observed, a *post-hoc* test was then performed) using Analyse-it Software for Microsoft Excel (Analyse-it Software Ltd, Leeds, United Kingdom). The presence of correlation between microbiota phylum and the absolute number of white blood cell classes was evaluated using the Spearmann's test. The r coefficient was interpreted according to Schober et al. (30). For all the statistical analyses, significance level was set at *P* < 0.05 (27).

## Results

### Dogs

Signalment of the 39 dogs enrolled in this study is summarized in Table 2. Based on clinical examination and screening analyses, dogs were divided into infected symptomatic (S, 12 dogs), exposed asymptomatic (E_A, 13 dogs) and healthy controls (H, 14 dogs). All the dogs belonging to S group presented with leishmaniasis clinical signs of disease, positive IFAT and a positive result for a direct test (qPCR or cytology) (Table 3). For dogs 11, 17 and 36 the amount of EDTA whole blood was not sufficient to perform the flow cytometric evaluation. Thus, Flow cytometry evaluation was finally performed on 36 dogs.

**Table 2:** Signalment and groups of the dogs enrolled in this study.

**Id**	**Group**	**Breed**	**Age**	**Gender**
1	H	Mixed-breed	1 YY 10 MM	MN
2	H	Medium Schnauzer	1YY 3 MM	FS
3	H	Poodle	1,5 YY	MN
4	H	Mixed-breed	7 YY	FS
5	H	Mixed-breed	1 YY	MN
6	H	CKCS	11 YY	MN
7	H	Golden Retriever	5,5 YY	MN
8	H	Mixed-breed	1,5 YY	FS
9	H	Australia shepherd	7 YY	FS
10	H	Mixed-breed	14 YY	FS
11	H	Border Collie	5,5 YY	FS
12	H	Mixed-breed	2YY 6 MM	MN
13	H	WHWT	4YY 11MM	FS
14	H	Border Collie	9YY	FS
15	E_A	Mixed-breed	8 YY	MN
16	E_A	Mixed-breed	6 YY	MN
17	E_A	Mixed-breed	6 YY	MN
18	E_A	Mixed-breed	4 YY	MN
19	E_A	Mixed-breed	14 YY	MN
20	E_A	Mixed-breed	4 YY	MN
21	E_A	Mixed-breed	1 YY	MN
22	E_A	Mixed-breed	1 YY	FS
23	E_A	Mixed-breed	1 YY	FS
24	E_A	Mixed-breed	1 YY	FS
25	E_A	Mixed-breed	10 YY 6 MM	MN
26	E_A	Rhodesian Ridgeback	10 YY	MN
27	E_A	Mixed-breed	8 YY	MN
28	S	Beagle	6 YY	FS
29	S	Mixed-breed	6 YY	FS
30	S	Mixed-breed	7 YY	FS
31	S	Golden Retriever	11 YY	M
32	S	Breton	4 YY	M
33	S	English Setter	3 YY	M
34	S	Mixed-breed	3,6 YY	F
35	S	Bloodhound	7 YY 8 MM	M
36	S	Labrador	7 YY	M
37	S	Mixed-breed	8 YY	F
38	S	Mixed-breed	2 YY	M
39	S	Mixed-breed	7 YY	M

*CKCS, cavalier king charles spaniel; WHWT, west highland white terrier; YY, years, MM, months, F, female, FS, female spayed, M, male, MN, male neutered*.

**Table 3:** Clinical signs and laboratory abnormalities of the dogs belonging to S group.

**Id**	**Clinical signs**	**SPE**	**IFAT**	**Direct tests**	**Lab abnormalities**
28	Periocular bilateral alopecia, pinnae bilateral crust	> α2	1:1,280	PCR	Mild leukopenia with reactive LGL, ↓alb, ↓A/G,
29	Alopecia	> γ	1:1,280	PCR, CYTOLOGY (spleen, BM)	Severe anemia normocytic normochromic and thrombocytopenia, mild leukocytosis with mature neutrophilia, ↓alb, ↓A/G,
30	Lymph node enlargement	–	1:160	PCR, CYTOLOGY (BM)	–
31	PU/PD, depression	> α2, β2, γ	1:640	PCR, CYTOLOGY (BM)	Mild normocytic normochromic anemia, severe proteinuria
32	Hyperthermia, lymph nodes enlargement, weight loss	> γ	>1:1,280	CYTOLOGY (LN)	Mild macrocytic hypochromic anemia, reactive LGL, ↑TP, ↓A/G,
33	Lymph nodes enlargement, hyperthermia, polyarthritis	> β2, γ	>1:1,280	PCR	Moderate macrocytic hypochromic anemia, lymphopenia, ↑TP, ↓alb, ↓A/G
34	Lameness and polyarthritis	> γ	1:5,120	PCR	Severe hypochromic anemia, ↑↑TP, ↓alb, ↓A/G, severe proteinuria
35	Lymph node enlargement anorexia, cachexia, pale mucous membranes.	> β2, γ	1:320	PCR, CYTOLOGY (LN)	Severe normocytic normochromic anemia, leukopenia, neutropenia, ↓alb, ↓A/G, ↑UREA, ↑CREA
36	Monolateral epistaxis	> α2, β2, γ	1:640	PCR	↑PT, ↓A/G
37	Mild depression	> α2, β2, γ	1:160	PCR, CYTOLOGY (LN)	Mild normocytic normochromic anemia, ↑TP, ↓A/G, mild proteinuria
38	Convulsion, bilateral nephropathy, urinary and bladder sediment.	> α2, γ	1:1,280	PCR	Severe thrombocytopenia, ↑↑UREA, ↑↑CREA, ↑TP, ↓alb, ↓A/G, ↑ALT
39	Dermatitis, PU/PD	> β2, γ	1:160	PCR	Leukocytosis with eosinophilia, reactive LGL, ↑TP, ↓A/G,

*BM, bone marrow; LN, lymph node; SPE, serum protein electrophoresis; IFAT, immunofluorescence antibody test; TP, total proteins; LGL, large granular lymphocytes; A/G, albumin to globulin ratio; alb, albumin; ALT, alanine aminotransferase; CREA, creatinine; PU/PD, polyuria/polydipsia*.

### Microbiota Analysis

**Figure 1:**
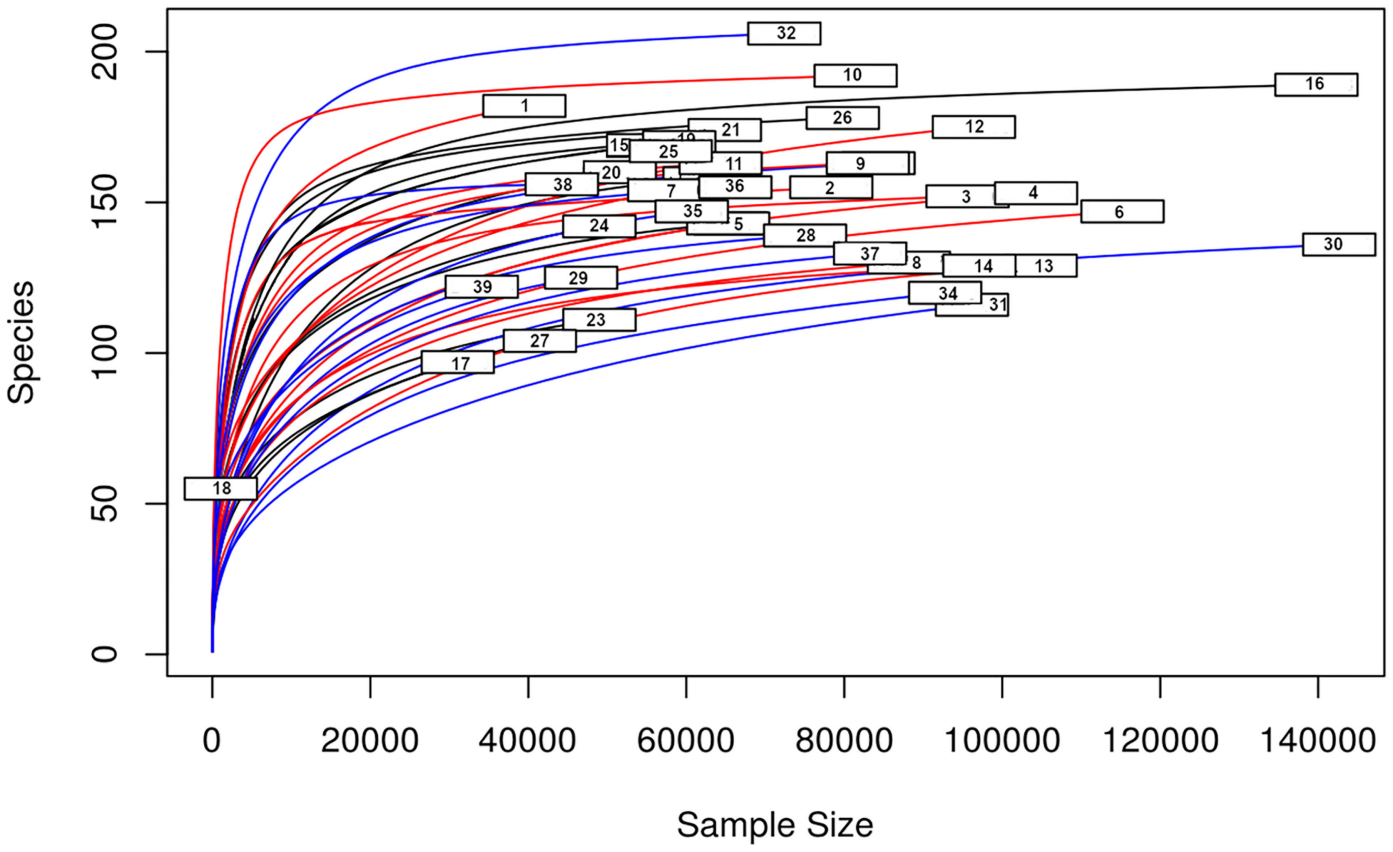
Alpha—diversity for the entire caseload. Each line represents one sample: in blue those belonging to S group, in red the H group, in black the E_A group. On the x-axis is reported the sequences number, the y-axis represented the number of different species.

**Figure 2:**
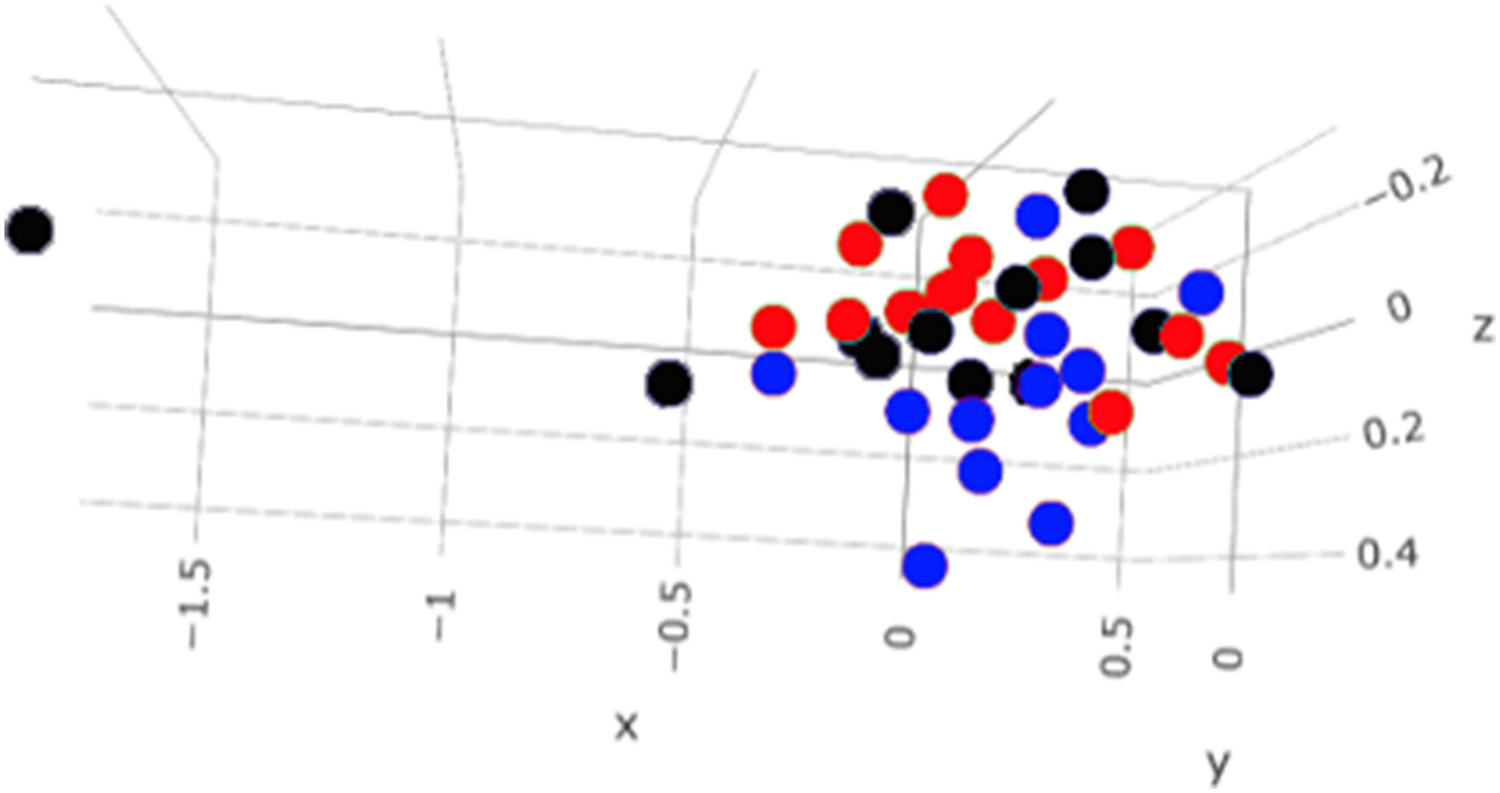
Beta—diversity of the entire caseload. Each dot represents one sample, in blue the symptomatic dogs (S), in red the healthy dogs (H) and in black the exposed asymptomatic (E_A). On each axis is reported the variance.

**Figure 3:**
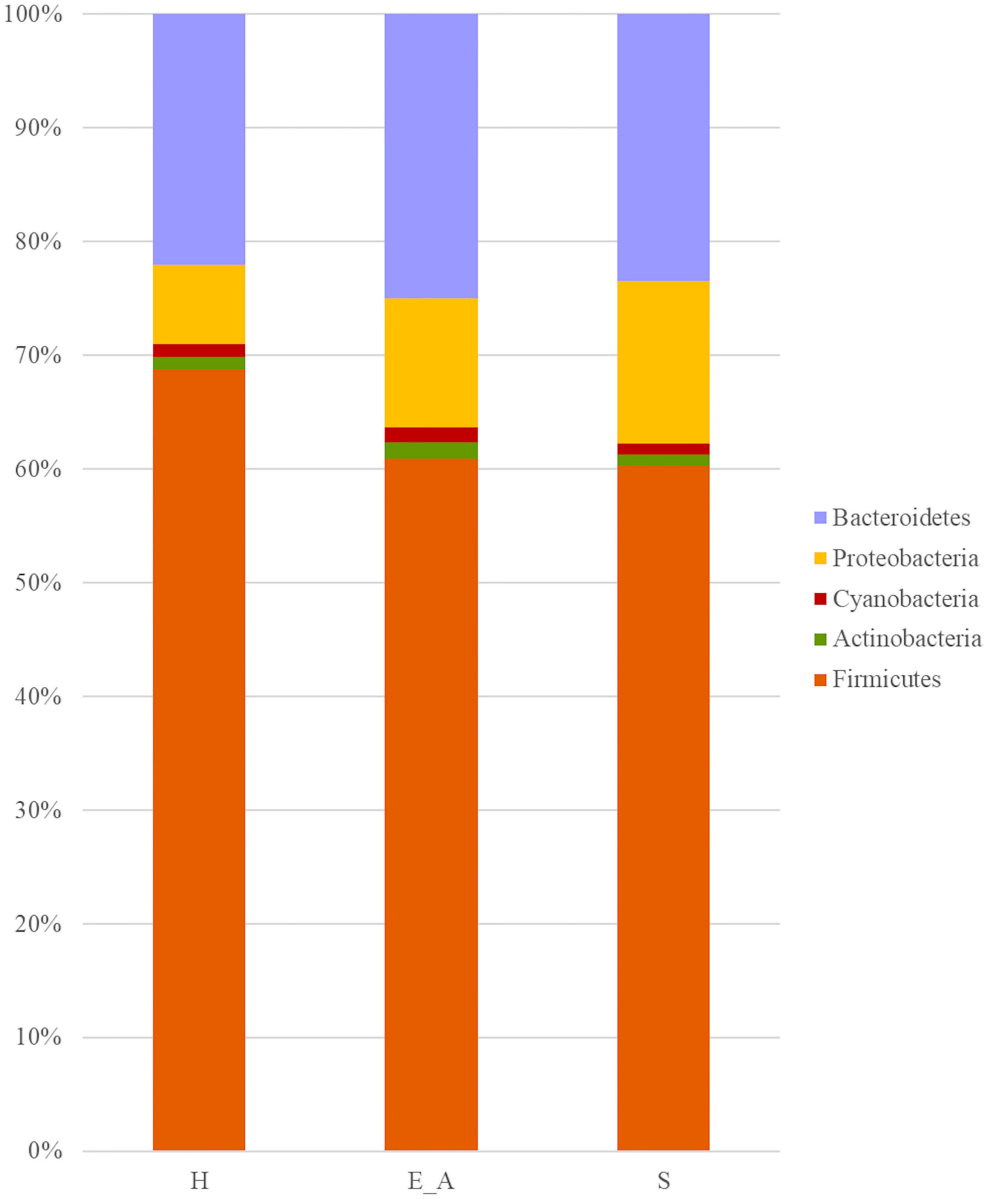
Bar plot representation of phylum relative abundance in the three groups. Only phylum with a relative abundance above 0.5% were reported in the graph.

**Figure 4:**
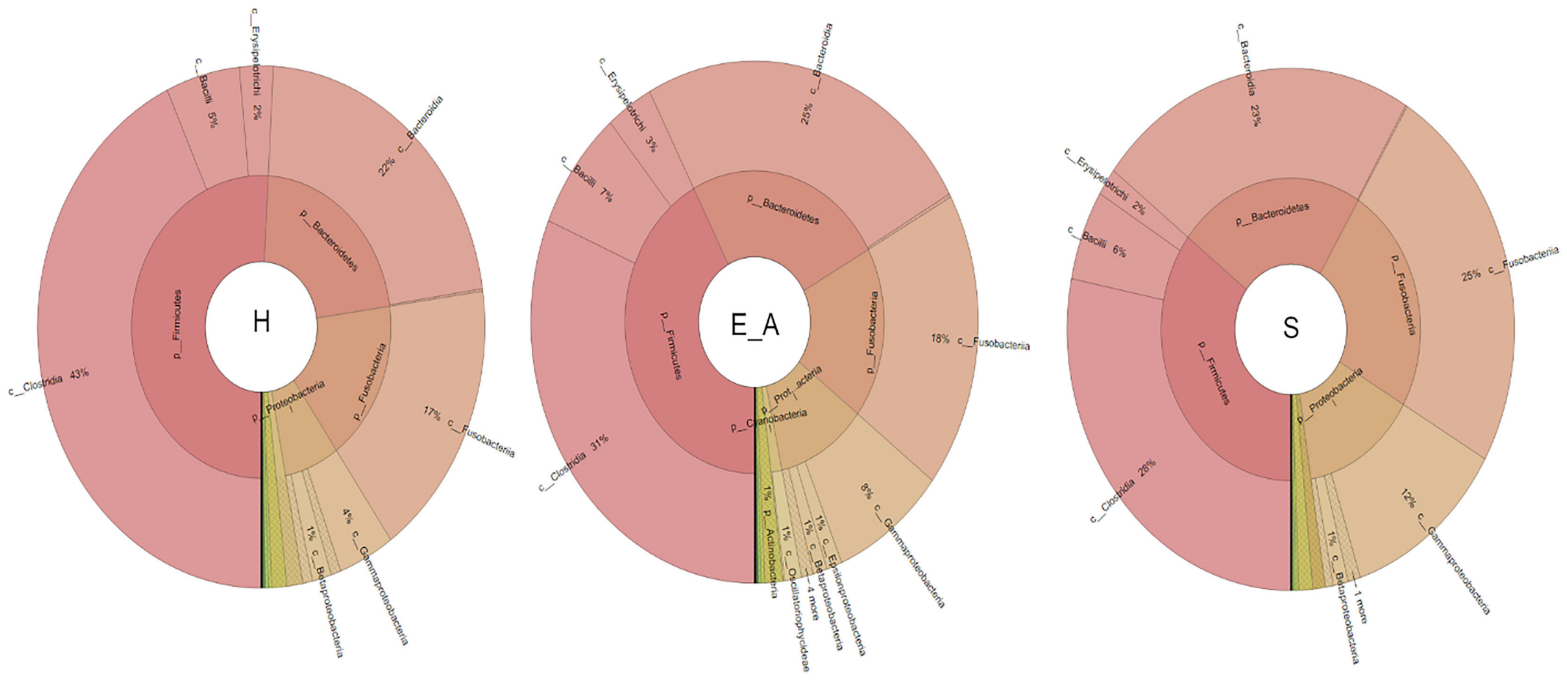
Microbiota composition of the three groups. Phyla and healthy classes of the bacteria sequenced in healthy (H), exposed asymptomatic (E_A) and symptomatic (S) dogs are represented through a Krona chart.

A total amount of 10,137,881 sequences, with a mean of 259,946 sequences/sample (median 273,190, range 69,776–273,190) were considered with an acceptable quality. The evaluation of alpha-diversity rarefaction curves confirmed the good quality of the entire caseload. Indeed, all the curves tended to grow very quickly and, in some cases, reached a plateau, consistent with a high richness of different bacterial species (Figure 1). Considering the beta-diversity (Figure 2), S group (blue dots), appeared to be grouped differently compared to the other groups (black and red dots that represent E_A and H, respectively) that seemed to be closer to each other. Firmicutes, Bacteroidetes, Proteobacteria, Actinobacteria and Cyanobacteria were the most represented phyla in the study population (Figure 3). According to the statistical analysis Firmicutes were more abundant in H group, than in E_A and S ones (*P* = 0.007 and =0.046, respectively). On the other hand, Proteobacteria was more represented in S than into H (*P* = 0.004). Significant differences were present even considering the rarest phyla: [Thermi] were less represented in E_A than into H group (*P* = 0.049), while Spirochaetes were less abundant in S than into H and E_A (*P* = 0.02 vs. H and =0.001 vs. E_A). Deferribacteres and Tenericutes were less represented in S than into the E_A group (*P* = 0.016 and =0.02, respectively). Class comparison showed that bacteria belonging to Firmicutes phylum such as Clostridia and Erysipelotrichi were more abundant in the H group, compared to the E_A (*P* = 0.03), and S group (*P* = 0.048). On the other hand, Gammaproteobacteria belonging to the Proteobacteria phylum was less abundant in H, compared to the other dogs (*P* = 0.043 in E_A and *P* =0.0003 in S). Significant differences were highlighted among groups even for rarest classes, Deinococci was less abundant in E_A compared to H dogs (*P* = 0.045), while [Brachyspirae], Deferribacteres and Mollicutes were more represented in E_A compared to S group (*P* = 0.007, =0.02 and =0.044, respectively) (Figure 4). Significant differences were observed even extending the analysis to order, family and genus (Tables 4, 5) (27).

**Table 4:** Relative abundances of the orders that showed significant differences among the groups.

**Order**	**H**	**E_A**	**S**
[Brachyspirales]	0.019	0.020a^*^	0.003a^*^
Aeromonadales	0.844a^*^	0.959b^*^	0.114a^*^, b^*^
Anaeroplasmatales	0.042	0.0245a	0.010a
Bifidobacteriales	0.026a^**^	0.007	0.011a^**^
Deferribacterales	0.044a^*^	0.034b	0.074a^*^, b
Enterobacteriales	2.722a^**^, b^**^	6.668a^**^	11.243b^**^
Erysipelotrichales	2.423	3.388a	1.704a
Lactobacillales	2.362a	4.897a	4.825
Pasteurellales	0.006a	0.030	0.021a
Streptophyta	0.010a	0.092a	0.018

*For each order the paired letters mean that a significant difference was found between groups. When no other symbol is present P < 0.05*.

* *P < 0.01 and*

** *P < 0.001*.

**Table 5:** Relative abundances of the families and genus that resulted significantly different among the three groups.

**Phylum**	**Family**	**H**	**E_A**	**S**	**Genus**	**H**	**E_A**	**S**
	*[o__Streptophyta]*	0.010a^*^	0.092a^*^	0.018				
Actinobacteria	*Actinomycetaceae*	0.006a	0.016b	0.072a, b	*Actinomyces*	0.006c^*^	0.016d	0.068c^*^, d
	*Bifidobacteriaceae*	0.026a^*^	0.007	0.011a^*^				
	*Micrococcaceae*	0.024a	0.015	0.006a				
					*Adlercreutzia*	0.058c	0.136d^**^	0.0243c, d^**^
Bacteroidetes	*Porphyromonadaceae*	0.114a^**^, b^**^	0.684a^**^	1.613b^**^	*Porphyromonas*	0.0005c, d^**^	0.108c	1.48d^**^
	*Prevotellaceae*	5.403a	10.224b^*^	0.557a, b^*^	*Prevotella*	5.403c	10.224d^*^	0.557c, d^*^
	*Flavobacteriaceae*	0.030a	0.067a	0.0324				
					*Parabacteroides*	0.096c^**^	0.557c^**^	0.115
Deferribacteres	Deferribacteraceae	0.044a^*^	0.034b	0.074a^*^. b	*Mucispirillum*	0.044c^*^	0.034d	0.074c^*^, d
Firmicutes	*[Tissierellaceae]*	0.127a	0.155	0.143a	*Allobaculum*	0.414	1.067c	0.276c
	*Clostridiaceae*	14.061a^**^	2.688a^**^, b^*^	6.974a^*^	*Clostridium*	13.549c, d^**^	2.414c, e^*^	6.603d^**^, e^*^
	*Enterococcaceae*	0.021a^*^	1.091	0.874a^*^	*Enterococcus*	0.020c^**^, d^*^	1.091c^**^	0.874d^*^
	*Erysipelotrichaceae*	2.423	3.388a	1.704a	*Holdemania*	0.023c	0.024	0.001c
	*Turicibacteraceae*	2.846a	2.433	0.657a	*Turicibacter*	2.846c^*^	2.433	0.657c^*^
	*Veillonellaceae*	2.810a^*^	1.961b^*^	0.809a^*^, b^*^				
					*Anaerofilum*	0.009c	0.009	0.109c
					*Catenibacterium*	0.628c^*^	0.680d^*^	0.0618c^*^, d^*^
					*Dorea*	1.624c^*^	2.034d^*^	3.659c^*^, d^*^
					*Megamonas*	2.214c^**^	1.222d^*^	0.189c^**^, d^*^
					*Peptostreptococcus*	0.001c^*^	0.006	0.024c^*^
					*Roseburia*	0.086c	0.029d^**^	0.276c, d^**^
					*Ruminococcus*	0.103	0.096c	0.202c
					*Streptococcus*	2.12	3.6228c	3.512c
					*Fusobacterium*	2.4635c	3454	5.584c
Proteobacteria	*Enterobacteriaceae*	2.722a^**^, b^**^	6.668a^**^	11.243b^**^	*Escherichia*	2.674c, d^*^	6.6285c	10.142d^*^
	*Hyphomicrobiaceae*	0.171	0.209a	0.130a				
	*Pasteurellaceae*	0.006a	0.030a	0.0210				
	*Succinivibrionaceae*	0.844a^*^	0.959b	0.114a^*^, b	*Anaerobiospirillum*	0.732	0.924c	0.103c
					*Mesorhizobium*	0c^*^	0.006c^*^	0.002
					*Phenylobacterium*	0.011c	0.001c	0.003
					*Proteus*	0.0002c^*^	0.001d	1.078c^*^, d
Spirochaetes	*Brachyspiraceae*	0.019	0.020a^*^	0.003a^*^	*Brachyspira*	0.019	0.020c^*^	0.003c^*^

*For each family and genus the paired letters mean that a significant difference was found between the groups with the same letter. When no other symbol is present P < 0.05*.

* *P < 0.01 and*

** *P < 0.001*.

### Comparison of Leukocyte Classes Among Groups

**Figure 5:**
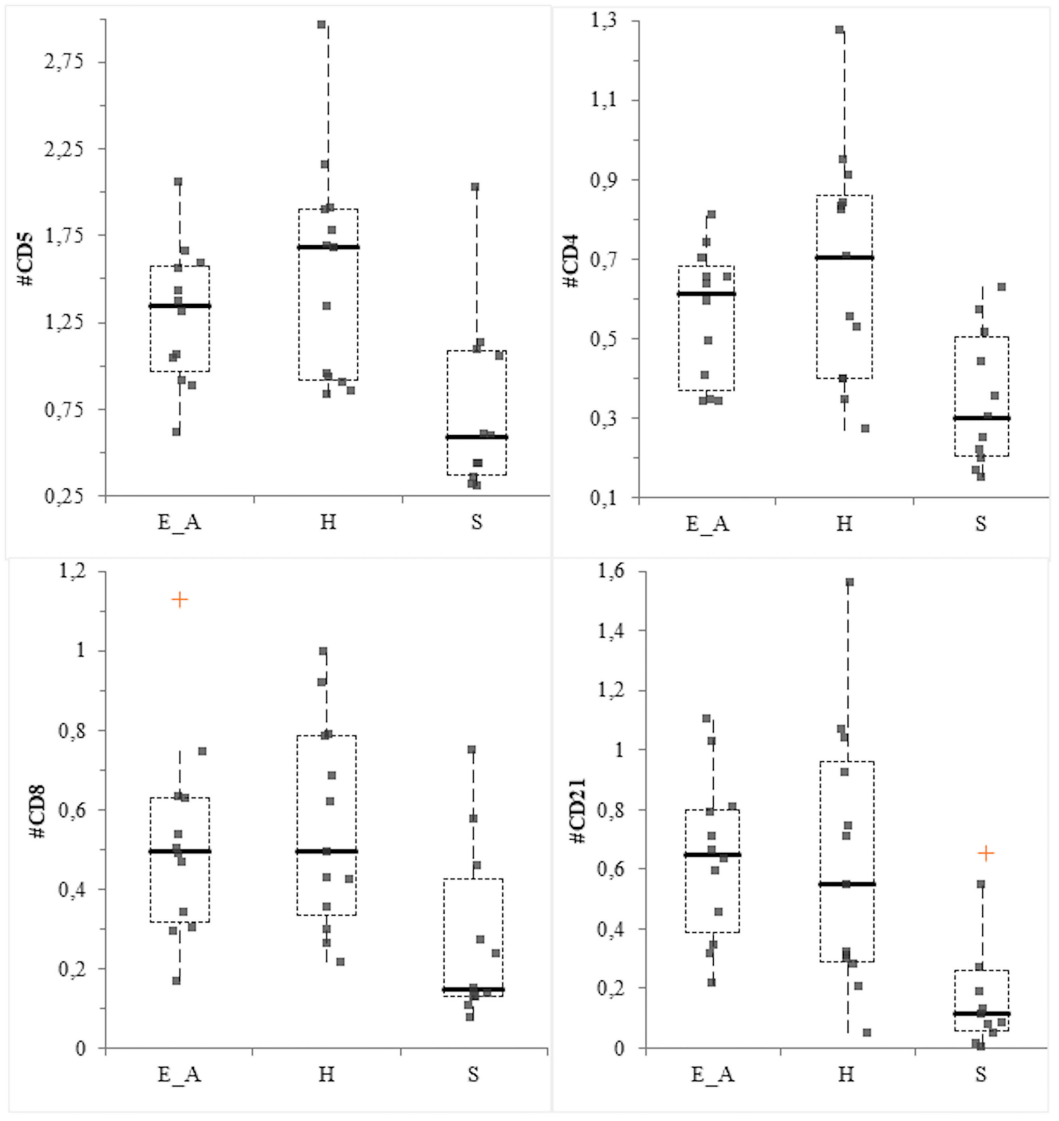
Box—plot for main lymphocyte classes in the three group of dogs. Comparison of the absolute value (expressed as ×103/μL) of CD5+ cells (upper left), CD4+ cells (upper right), CD8+ (lower left) and CD21+ (lower right) cells among the three group. The orange crosses represent the near outliers.

The CD14+ percentage and the CD4:CD8 ratio did not vary among groups (*P* = 0.56 and *P* =0.36, respectively). Conversely, significant differences were recorded for CD11b+ (*P* = 0.0026), neutrophils (*P* = 0.014), CD5+ (*P* = 0.0085), CD4+ (*P* = 0.032), CD8+ (*P* = 0.027) and CD21+ (*P* = 0.004) percentages among groups. Specifically, the percentage of CD11b and neutrophils was decreased in the H group compared to the E_A (*P* = 0.034 for both class of leukocytes) and to the S group (*P* = 0.001 for both class of leukocytes). The comparison between these latter two groups did not result in significant differences. The percentage of CD21+ and CD8+ cells was significantly decreased in the S group compared to E_A (*P* = 0.004 and 0.048, respectively) and H (*P* = 0.004 and = 0.012), while these two latter groups were not significantly different. The percentage of CD4+ and CD5+ was significantly decreased in the S group, only compared to the H group (*P* = 0.008 and *P* = 0.004, respectively), but not to the E_A one. The absolute number of CD14+, CD11b+ and neutrophils did not vary among groups (*P* = 0.72, *P* = 0.6, and *P* = 0.57, respectively). Conversely, CD5+ (*P* = 0.0097), CD4+ (*P* = 0.0047), CD8+ (*P* = 0.0106) and CD21+ (*P* = 0.0011) were significantly different. Specifically, all these lymphocyte classes were decreased in the S dogs compared to E_A (*P* = 0.012, 0.008, 0.012, and 0.003, respectively) and H (*P* = 0.008, 0.005, 0.007, and 0.0007, respectively). However, these latter groups were not significantly different for any of the lymphocyte classes (Figure 5).

### Correlation Between Microbiota Phyla and Leukocyte Classes

The correlation analysis between gut microbiota phyla relative abundance and the leukocyte classes absolute number showed a mild positive correlation between Actinobacteria and CD11b (*r* = 0.35) and between Fusobacteria and CD4:CD8 ratio (*r* = 0.33). A mild negative correlation was observed between Fusobacteria and CD11b (*r* = −0.35) and neutrophils (*r* = −0.39).

## Discussion

To the authors knowledge this study is the first reporting results about possible correlations between gut microbiota and leukocytes subclasses in dogs with clinical signs of *L. infantum* infection, exposed asymptomatic dogs and healthy unexposed controls. The study of microbiota is a field of great interest in both human and veterinary medicine, due to both the relationship with immunity and the possible therapeutic application (31). The results obtained in this study, showing that the phyla most represented in healthy dogs were Firmicutes, Bacteroidetes, Actinobacteria and Proteobacteria, agree with previous studies (32). Specifically, Firmicutes phylum has been frequently found as more represented in healthy animals, both in literature and in this study, as well as two of its main represented classes, Clostridia and Erysipelotrichi (33). Interestingly, the result obtained in this study regarding Proteobacteria phylum, more abundant in symptomatic dogs than into the healthy ones, is in accordance with previous reports (14, 34). It should be reminded that Proteobacteria phylum, that includes several gastrointestinal pathogens (e.g., *Escherichia coli*), is of particular concern in veterinary medicine. Thus, this finding in symptomatic dogs may suggest the presence of a subtle dysbiosis even in dogs that do not clearly show clinical signs related with a gastrointestinal disease. Moreover, the significant higher abundance of Gammaproteobacteria, which is the main class belonging to Proteobacteria phylum, as well as other bacteria belonging to genera of this phylum (specifically *Enterococcus* and *Proteus*) in both symptomatic and asymptomatic dogs, compared to the healthy ones, may also suggest the presence of dysbiosis in infected dogs. The significant results at the genus level showing a lower abundance of *Mucispirillum* (Fam. *Deferribacteraceae*, phylum Deferribacteres) in dogs with clinical signs of the disease compared to the other two groups needs further investigations. In mice gut microbiota *Mucispirillum* has been reported to be protective against Salmonella infection (35). There is no information about the role of this genus in dogs, but the lower abundance recorded in symptomatic dogs may suggest the presence of dysbiosis or of gastrointestinal increased susceptibility to diseases. Overall, most of the less represented families and genera in the symptomatic group (*Veillonellaceae, Megamonas, Prevotella, Catenibacterium* and *Clostridium*) belong to the Firmicutes and Bacteroidetes phyla and are related with the short chain fatty acids (SCFAs) production. These metabolites (specifically acetate, propionate and butyrate), that derive from simple carbohydrates fermentation, are reported to have a main role in the enterocyte's nutrition and in immunomodulation. Indeed, it seems that they are associated with the production enhancement of anti-inflammatory cytokines (e.g., IL-10 and TGFβ) and the proinflammatory cytokines suppression (e.g., IL-6, IL-8 and TNFα) (36, 37). It could be interesting in future studies to investigate whether symptomatic dogs are associated to a decrease of SCFAs synthesis through a metabolomic approach. Differences between *Leishmania*-infected dogs (both with or without clinical signs) and healthy dogs were not surprising. Indeed, an alteration of gut microbiota composition in dogs with disorders other than gastrointestinal diseases (e.g., multicentric lymphoma, diabetes mellitus) has already been reported (15, 38). However, literature about healthy or diseased microbiota composition in dogs is not concordant. Moreover, attention should be driven also to factors other than the presence of diseases or infections that may influence the microbiota composition. In the present study, in order to minimize possible confounding factors, all the enrolled dogs were fed on commercial food only. However, the extent of the dietary influence on the caseload of this study is not completely known, since there is some variability in the type of commercial food. In addition, environment, age and body condition score may also influence the microbiota, as well as the individual variability that may be present in the healthy population. Thus, a larger study group may help in confirming results obtained here.

In this study, results related to the leukocyte populations agree with some previous studies reporting the pivotal role of the immune system in the evolution and clinical results of *Leishmania* spp. infection in dogs (16). In the cutaneous leishmaniasis murine model, resistance to the disease has been correlated with a prevalent Th1 response, while susceptibility has been associated with a predominant Th2 response alone. In canine leishmaniasis, a major protection is associated with a Th1-like immunity (39). In the present study all the lymphocyte subsets were significantly lower in the dogs with clinical signs compared with both the exposed asymptomatic and the healthy ones. Even though B-cells are implicated in the plasma cells activation and production of immunoglobulin, cause of canine leishmaniasis symptomatic form of disease (17), this stimulation usually takes place in lymphoid organs or in bone marrow. Thus, it is not astonishing to observe a decrease (both in percentage and absolute number) of circulating CD21+ cells in symptomatic dogs, as already reported (22, 40). Since the decrease of T lymphocytes associated with a Th1 response is related with the disease susceptibility (22, 40) the significantly lower amount of CD5+ and CD4+ cells in the symptomatic group are expected as well. The results obtained from the evaluation of CD4:CD8 ratio in dogs affected by *L. infantum* infection are controversial. Some studies reported a decrease of this ratio in dogs with clinical signs of leishmaniasis (41, 42). However, in the present study a significant difference in CD4:CD8 was not found. This result may be associated with a decrease of both CD8+ and CD4+ cells in symptomatic dogs that subsequently led to an absence of variation of the ratio. The visual inspection of the box plot graph suggests that the lowest results for CD4:CD8 were observed in the asymptomatic group. This evidence confirms what reported by Reis et al. (22) where a CD8+ cells increase is described to cause an inversion of CD4:CD8 ratio, in relationship with a low parasite burden in the bone marrow. The different results reported previously likely reflect the complexity of this disease, thus a further investigation about the role of Th1/Th2 response in the pathogenesis of the disease could be interesting, for example by evaluating cytokine expression in canine patients. The results of the correlation between phyla abundance and leukocyte classes, even though statistically significant, were not robust enough to be relevant, being r values lower than 0.4.

One limitation of this study is represented by the small sampling population, thus the results here obtained should be confirmed on a larger number of dogs. Anyway, it should be pointed out that investigations on canine microbiota during systemic infectious diseases are still scarce in literature, thus results here observed still have an important role in gathering information about this topic in canine medicine. Another limitation of this study concerns the inclusion of exposed asymptomatic, instead of infected asymptomatic dogs. This choice was due to ethical issues, since a positive PCR result on a bone marrow sample should have been obtained to confirm hidden infection. Unfortunately, bone marrow sampling is a quite invasive procedure requiring at least local anesthesia, even when performed by experienced clinicians, making it not be ethically advisable in clinically healthy dogs. For this reason, in this study, qPCR was performed on leftover whole blood specimens that had been sampled for diagnostic purposes or routine health check. In literature, some papers report good sensitivity and specificity for qPCR on whole blood, also in asymptomatic dogs (43). Nevertheless, results obtained in this study on the exposed asymptomatic group should be confirmed in infected asymptomatic dogs. Another possible limitation is the inclusion of all the symptomatic dogs in one group despite different clinical presentations of the disease. In this study symptomatic dogs were enrolled according to Canine Leishmaniasis Working Group guidelines (17). However, the Leishvet group guidelines allow to further classify the sick dogs based on the severity of clinical signs (44). Thus, it would be interesting to investigate in future studies if the difference here observed in canine gut microbiota of sick dogs would be confirmed even with different clinical presentation of the disease. In conclusion, the gut microbiota composition in dogs with or without *Leishmania* spp. infection was significantly different among groups, mostly in the Firmicutes and Proteobacteria phyla. This observation agrees with previous studies about systemic or local gastrointestinal diseases other than leishmaniasis in dogs (15, 45). The investigation on a higher number of cases, possibly including infected dogs without clinical signs, rather than exposed dogs, could confirm the results obtained in this study. To the authors' knowledge this study is the first that investigate the gut microbiota composition in association with canine leishmaniasis, providing new insights on this disease. Moreover, a recent study on a hamster model for fatal visceral leishmaniasis reported how the dysbiosis, induced by a long-term antibiotic treatment, seems to delay the onset and reduce the severity of the disease, suggesting that a modification in microbiota composition could possibly affect the disease development (46). These results, together with those obtained in the present study, should further spur the researcher into investigation on canine gut microbiota during leishmaniasis as a possible target of intervention to modify the disease development. Flow cytometric results partially agree with those reported in literature, showing a decrease of all the lymphocytes classes in symptomatic dogs. However, it would be interesting to deeply investigate the role of immunity analyzing the pattern of cytokine expression in the different groups. The preliminary results obtained on the correlation between leukocyte classes and microbiota composition need to be confirmed on a higher number of dogs. Moreover, due to the absence of data about this topic, it is quite difficult to provide a biological explanation, even though these preliminary results may be a starting point for further analyses.

## Data Availability Statement

The original contributions presented in the study are publicly available. This data can be found here: https://www.ncbi.nlm.nih.gov/bioproject/PRJNA837097.

## Ethics Statement

Samples from all the dogs were collected according to a protocol approved by the Ethical Committee of the University of Milan (protocol no. 02-2016) that allow the use of residual samples and tissues collected for routine or diagnostic purposes with the informed consent of the owners, without any further formal request or authorization.

## Author Contributions

AG and SM contributed to conception, design of the study, and wrote the first draft of the manuscript. MP, SZ, SM, SL, RF, and VM organized the database. SM performed the statistical analysis. AG, SM, SZ, SL, and VM wrote sections of the manuscript. All authors contributed to manuscript revision, read, and approved the submitted version.

## Conflict of Interest

The authors declare that the research was conducted in the absence of any commercial or financial relationships that could be construed as a potential conflict of interest.

## Publisher's Note

All claims expressed in this article are solely those of the authors and do not necessarily represent those of their affiliated organizations, or those of the publisher, the editors and the reviewers. Any product that may be evaluated in this article, or claim that may be made by its manufacturer, is not guaranteed or endorsed by the publisher.
